# Investigating Brain MRI Findings in Children with Headache

**Published:** 2018

**Authors:** Abdulrasool AlAEE, Ali ABBASKHANIAN, Maedeh AZIMI, Mohammadreza AZIMI

**Affiliations:** 1 Department of Radiology, Mazandaran University of Medical Sciences, Sari, Iran; 2 Department of Pediatric Neurology, Faculty of Medicine, Mazandaran University of Medical Sciences, Sari, Iran; 3 Department of Radiology, Iran Army University of Medical Sciences, Tehran, Iran.

**Keywords:** Children, Migraine, Headache, White matter hyperintensity, Brain, MRI

## Abstract

**Objective:**

Migraine is a common headache associated with structural changes in brain. The purpose of this study was to evaluate brain MRI findings in children with migraine.

**Materials & Methods:**

This cross-sectional study was conducted at Booali Hospital, Mazandaran University of Medical Sciences, Sari, Iran. Participants with headache and age between 5 and 15 yr were evaluated with MRI and their headache type was diagnosed by the standard criteria. The findings of the MRI were interpreted by a radiologist blinded to the diagnoses.

**Results:**

Overall, 81 individuals with symptom of headache and the mean age of 9.56±3.25 yr were enrolled. Twenty patients with the mean age of 9.65±2.75 yr were diagnosed with migraine without aura. Among the 54 male patients, 8 patients (14.8%) were diagnosed with migraine; and among the 27 female patients, 12 patients (44.4%) were diagnosed with migraine (RR: 1.5, 95%CI: 1.07-2.18, *P*=0.004). Ten migraine patients had abnormal MRI findings (50%), including 8 cases with high signal white matter lesion, and 2 cases with empty sella. The occurrence of the high signal white matter lesions was significantly greater in the migraine patients (RR: 3.91, 95% CI: 2.10-7.25, *P*=0.001).

**Conclusion:**

The possibility of occurrence of the high signal white matter lesions in the brain MRI of children with migraine was significantly higher compared with other headache types.

## Introduction

Headache is common in children and adolescents. National Health Interview Survey (NHIS) has reported that the prevalence of severe and recurring headaches was 25.3 per 1000 persons, aged ˂18 yr, and 9.9 per 1000 persons, aged ˂ 10 yr (1). Migraine is the most common causes of a recurrent headache in more than half of children and adolescent. This type of headache has high prevalence in school-age children and significantly affected on their school attendance and family quality of life (2). 

Migraine is manifested by periodic, unilateral and pulsating headache which typically last between 4 and 72 hour. Migraine may be associated with neurological, digestive and autonomic symptoms. Migraine may be divided into 2 types: classic migraine called migraine with aura and common migraine called migraine without aura (2, 3).

The primary medical concern over children presenting with headaches is the probability of intracranial pathology. Due to the increased number of brain imaging centers, the parents` demand for radiological evaluation has increased (4). Moreover, it is difficult for physicians to distinguish between first, second, and third migraine headaches attacks and headaches caused by brain tumors, subarachnoid hemorrhage, vasculopathy, arteriovenous malformation, and other underlying diseases (5). These patients usually undergo brain radiological evaluation before diagnosed migraine. However, the physicians do not recommend radiological evaluation for a migraine with typical patterns (6). 

The case of complicated migraine with focal neurological signs whose manifestations cannot be distinguished from the manifestations of intracranial neoplasm, brain imaging is recommended (2). In addition, imaging is recommended for ophthalmoplegic migraine, hemiplegic migraine, and basilar migraine (involvement of brain nerve 3, 4, 6) (2). Brain imaging finding in children with headaches especially migraine included sinusitis, Chiari malformation type I, unspecified white matter changes, venous angioma, arachnoid cyst, pineal cyst, mega cisterna magna, and malignant cases (7). Moreover, these abnormal results can be obtained with healthy controls, as random results. For instance, white matter lesions can be seen in healthy individuals´ MRI who had risk factors for cerebral vascular diseases (8). 

Therefore, this study was conducted to investigate MRI findings in children with headache.

## Materials & Methods

**Patients and design:**

This cross-sectional study investigated children with headaches at Mazandaran University of Medical Sciences, Mazandaran, Iran between 2014 and 2015. Inclusion criteria were: all patients with headache, aged between 5 to 15 yr. Our exclusion criteria included: all patients with already certain neurological defects or history of abnormalities in MRI, patients with underlying diseases (collagen vascular, hypertension, diabetes, right to left shunt, and cardiac valvulopathy), patients with a past history of having cerebral ischemic disease, cerebral demyelination, and central nervous system vasculitis.

Based on the literature and all children with headache who presented to primary children’s medical center that 81 individuals were invited to participate (9). 

Approval for this study was obtained from the Investigational Review Board at our institution (code: 91-189).

**Data collection:**

All participants underwent brain MRI (GE-1/5 Tesla) and through multi-planar method in FLAIR (fluid-attenuated inversion recovery) sequence. Next, the results were interpreted by an experienced radiologist. The type of headache was determined by a neurologist, based on diagnostic criteria. Finally, migraine patients´ MRI results were studied and compared with other patients (10).

**Statistical analysis:**

Statistical analyses were conducted using SPSS (ver. 16, Chicago, IL, USA). Qualitative data were analyzed by Chi-square test, and Fisher´s exact test was used when it was required. Moreover, quantitative data were analyzed by *t*-test and X2. A *P*-value of ˂0.05 was considered statistically significant.

**Results**

Eighty-one patients with symptom of headache with the mean age of 9.56±3.25 yr (median=9) were enrolled. Overall, 54 of the participants were male (66.7%). Nineteen patients (23.5%) experienced unilateral headaches. Sixteen patients (19.8%) experienced throbbing headaches. Twenty-five patients (30.9%) reported that their headache was connected with their activities. Moreover, 40 patients (49.4%) had nausea along with headache. However, only 10 patients (12.3%) had vomiting. photophobia, phonophobia, and photopsia were reported by 9 patients (11.1%), 12 patients (14.8%), 10 patients (12.3%), respectively. Moreover, 2 patients (2.5%) experienced paresthesia. None experienced lethargy, dysphonia, or phantosmia.

The most prevalent diagnoses of headaches were migraine and sinusitis affecting 20 patients (24.7%) and 19 patients (23.5%), respectively (Table 1).

**Table 1:** Final diagnosis by neurologist

Type of headache	Frequency (%)
Unspecific headache	16 (19.8)
Migraine	20 (24.7)
Sinusitis	19 (23.5)
Tension headache	12 (14.8)
R/O SOL	7 (8.6)
Eye refractory error	7 (8.6)

R/O SOL: Rule out space occupying lesion.

Forty-one patients´ MRI results (50.6%) were abnormal. The most common abnormalities were: 14 patients (17.3%) with pansinusitis, 8 patients (9.9%) with high signal lesions, and 6 patients (7.4%) with empty sella (Table 2).

**Table 2:** MRI findings in all patients

MRI finding	Frequency (%)
Normal	40 (49.4)
Pansinusitis	14 (17.3)
High signal white matter lesion	8 (9.9)
Empty Sella	6 (7.4)
Sphenoiditis	4 (4.9)
Ethmoiditis	3 (3.7)
Tumor	3 (3.7)
Mastoiditis	2 (2.4)
Enlarged cisterna magna	1 (1.2)

**Migraine patients´ characteristics:** Diagnosis of migraine without aura was made by the pediatric neurologist in according to the international headache society criteria for pediatric migraine. The mean age of the migraine patients and other patients were 9.54±2.75 yr (median=9.5) and 9.54±3.42 yr, respectively (*P*=0.89). Eight (14.8%) out of 54 male cases and 12 (44.4%) out of 27 female cases were diagnosed with migraine. The prevalence of migraine was significantly higher in the females (1.5) compared with males (RR: 1.5, 95% CI: 1.07-2.18, *P*=0.004) (Table 3).

**Table 3:** Migraine characteristics

Symptoms	Percentage
Throbbing headache	95
Headache with vomiting	95
Headache > 3hr	90
Unilateral headache	80
Activity-induced headache	55

The prevalence of these symptoms was significantly higher among the migraine patients compared with the other patients (*P*˂0.001). The possibility of having migraine in those having 3 or 4 out of the 5 aforementioned symptoms were 21.33 (RR: 21.33, 95% CI: 6.02-300.6, *P*˂0.0001) and 42.56 (RR: 42.56, 95% CI: 6.02-300.6, *P*˂0.0001), respectively. However, the possibility of having migraine in the patients having 1 or 2 out of the 5 aforementioned symptoms was 0.23 (RR: 0.23, 95% CI: 0.16-0.35, *P*=0.079).* Relative risk *is the ratio of the probability of migraine attack in first group to the probability of a migraine occurring in a comparison, second group.

There were no significant differences in number of attacks per month (7.05±4.14 *vs.* 12.67±13.13) and duration of the attacks per hours (39.5±32.11 *vs.* 46.48±67.49) between migraine and non-migraine patients (*P*>0.05). 

Among the 19 patients with a family history of migraine, 17 patients (89.5%) were diagnosed with migraine. In the patients with a family history of migraine, the relative risk of being affected by migraine was 17.6 compared with the other participants (RR: 17.6, 95% CI: 5.78-53.56, *P*˂0.0001).

**Fig. 1:**
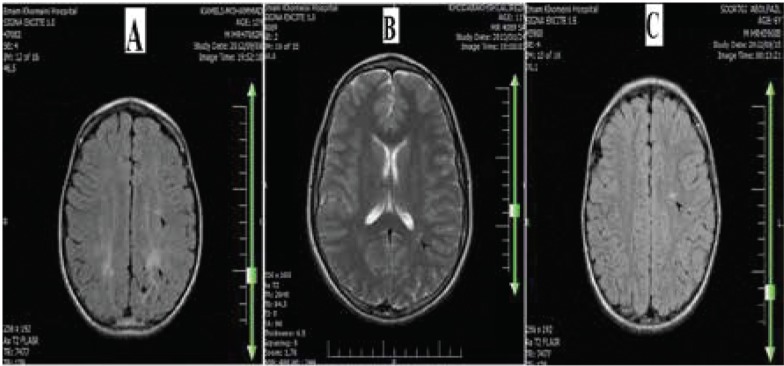
High signal areas in the periventricular white matter near the trigonearea of the lateral ventricles and semiovale centrum (A) periventricular white matter near the trigone area (B) semiovale centrum (C) T2 FLAIR MRI

Ten patients (50%) had abnormal MRI findings. These abnormalities were: 8 patients (80%) with high signal areas (3 patients in the frontal periventricular area, 2 patients in semiovale centrum, 2 patients in the periventricular white matter near the trigone area of the lateral ventricles and 1 patient with abnormalities in both the periventricular white matter near the trigone area of the lateral ventricles and in the semiovale centrum) and 2 patients (20%) with empty sella (Figure 1). The possibility of high signal white matter lesions in the brain MRI of migraine patients was 3.91 fold higher than other patients (RR:3.91, 95% CI:2.10-7.25, *P*=0.001).

## Discussion

Migraine is a chronic, multifactorial neurovascular disorder typically characterized by recurring and disabling headache attacks and dysfunction of autonomic nervous system (11). The prevalence of migraine in general population is 15% (12) and it has a cumulative lifetime incidence of 43% in women and 18% in men (13). Migraine causes pain, disability and decreased overall quality of life (14, 15). In the USA and Europe, the direct and indirect medical costs from migraine exceed $ 15 billion and € 27 billion per yr, respectively (16, 17). A better investigation of migraine mechanisms will result in more effective treatments and reduction of migraine impact (18). Thereby, this study designed to investigate brain MRI findings in children with migraine. 

The prevalence of migraine among the children presented with headache was 23.5%. The overall prevalence of migraine was 16.72% among the children with headache (19). In general, the overall prevalence of migraine among the children and adolescents with headache was 1.7%-21.4% (19). In our study, same as another study, the prevalence and relative risk of migraine was greater in females compared with males (20).

Our results revealed that in the patients having 4 out of the 5 aforementioned symptoms (headache lasting for 4-72, throbbing headache, unilateral headache, headache associated with vomiting, and headache worsened by activity), the possibility of being affected by migraine was 21.33 .

In the patients with 1 or 2 out of these symptoms, the possibility of being affected by migraine was 0.23. In a similar with our study, in the patients who had 4 out of the 5 aforementioned symptoms, the possibility of being affected by migraine was 24 (RR: 24, 95% CI: 1.5-388), and in the patients who having 1 or 2 out of these symptoms, the possibility of being affected by migraine reduced to 0.41 (RR: 0.41, 95% CI: 0.32-0.52) (21).

Several studies reported various brain lesions such as deep white matter lesions, stroke, and increased iron accumulation in migraine patients (2,11,18). In this study, the MRI findings of the 50% of the migraine patients were abnormal. The most common abnormalities were the high signal white matter lesions and the occurrence of these lesions was significantly higher in the migraine patients compared with other participants. On the contrary, the high signal white matter lesions were only seen in 17% of the children with migraine (22). Whereas same as our study, the MRI findings of 55% of the children with migraine were abnormal, but the abnormalities were not in concordance with our results. They had diagnosed 2 patients with asymmetry of lateral ventricles, 2 patients with demyelination, 2 patients with CSF space enlargement, and 2 patients with vascular malformation (23). The prevalence of white matter lesions was 10% in pediatric patients with migraine (without aura) and 4% in control group (*P*=0.119) (24). The high signal white matter lesions were reported in 10.6% of the children with migraine while the healthy children had no high signal white matter lesions (25). The high signal white matter lesions were reported in 50% of the children with migraine and in 17% of the control group (26). Contrary to the studies showed that the prevalence of high signal white matter lesions among the children with migraine was significantly high (22-26). In addition, the studies on adults with migraine showed that the prevalence of the high signal white matter lesions was between 12%-47% in migraine cases compared with normal cases (27-30). The risk of the high signal white matter lesions in the migraine patients was 3.91 in our study and 2.1 on adults (95% CI: 1.0-4.1, *P*=0.04) (31). A meta-analysis of 7 studies showed that the risk of the high signal white matter lesions in the migraine patients was 3.9 (95% CI: 2.26-6.72) compared with the general population; which was in close concordance with our results (32). In addition, the prevalence of these lesions was significantly higher in women with increased headache attacks (11). However, such results of the studies on adults were not in concordance with our results. Our study showed that there was no significant difference between males and females in the prevalence of the high signal white matter lesions.

**In conclusion,** the number of high signal white matter lesions in the brain MRI of children with migraine was significantly higher compared with other headaches. Moreover, there was no significant difference between males and females in the prevalence of these lesions.

## References

[1] Newackeck PW, Taylor WR (1992). Childhood chronic illness: prevalence, severity and impact. Am J Public Health.

[2] Shah UH, Kalra V (2009). Pediatric migraine. Int J Pediatr.

[3] Gutiérrez-Mata AP, López-Casas J, Ortez-González CI (2008). Clinical characteristics and progress of patients with migrainous headaches monitored in the Headache Unit in a paediatric referral hospital. Rev Neurol.

[4] Gozke E, Ore O, Dortcan N, Unal Z (2004). Cranial magnetic resonance imaging findings in patients with migraine. Headache.

[5] Iurlaro S, Beghi E, Massetto N (2004). Does headache represent a clinical marker in early diagnosis of cerebral venous thrombosis? A prospective multicentric study. NeurolSci.

[6] Chu ML, Shinnar S (1992). Headaches in children younger than 7 years of age. Arch Neurol.

[7] Schwedt TJ, Guo Y, Rothner AD (2006). "Benign" imaging abnormalities in children and adolescents with headache. Headache.

[8] Porter A, Gladstone JP, Dodick DW (2005). Migraine and white matter hyperintensities. Curr Pain Headache Rep.

[9] Xiang J, Degrauw X, Korman AM, Allen JR, O'Brien HL, Kabbouche MA (2013). Neuromagnetic abnormality of motor cortical activation and phases of headache attacks in childhood migraine. PLoS One.

[10] Kliegman R M, Stanton B F, St Geme JW, Schor NF (2016). Nelson Textbook of Pediatrics.

[11] Kruit MC, van Buchem MA, Hofman PA (2004). Migraine as a risk factor for subclinical brain lesions. JAMA.

[12] Sprenger T, Borsook D (2012). Migraine changes the brain: neuroimaging makes its mark. Curr Opin Neurol.

[13] Stewart WF, Wood C, Reed ML (2008). Cumulative lifetime migraine incidence in women and men. Cephalalgia.

[14] Terwindt GM, Ferrari MD, Tijhuis M (2000). The impact of migraine on quality of life in the general population: the GEM study. Neurology.

[15] Lipton RB, Hamelsky SW, Kolodner KB (2000). Migraine, quality of life, and depression: a population-based case-control study. Neurology.

[16] Hu XH, Markson LE, Lipton RB (1999). Burden of migraine in the United States: disability and economic costs. Arch Intern Med.

[17] Andlin-Sobocki P, Jonsson B, Wittchen HU (2005). Cost of disorders of the brain in Europe. Eur J Neurol.

[18] Schwedt TJ, Dodick DW (2009). Advanced neuroimaging of migraine. Lancet Neurol.

[19] Wöber-Bingöl C (2013). Epidemiology of migraine and headache in children and adolescents. Curr Pain Headache Rep.

[20] Abu-Arafeh I, Razak S, Sivaraman B (2010). Prevalence of headache and migraine in children and adolescents: a systematic review of population-based studies. Dev Med Child Neurol.

[21] Michel P, Henry P, Letenneur L (1993). Diagnostic screen for assessment of the IHS criteria for migraine by general practitioners. Cephalalgia.

[22] Candee MS, McCandless RT, Moore KR (2013). White matter lesions in children and adolescents with migraine. Pediatr Neurol.

[23] Zajac A, Herman-Sucharska I, Kubik A (2007). MRI and MRA data in children with migraine with aura. PrzeglLek.

[24] Mar S, Kelly JE, Isbell S (2013). Prevalence of white matter lesions and stroke in children with migraine. Neurology.

[25] Eidlitz-Markus T, Zeharia A, Haimi-Cohen Y (2013). MRI white matter lesions in pediatric migraine. Cephalalgia.

[26] Hämäläinen ML, Autti T, Salonen O (1996). Brain MRI in children with migraine: a controlled morphometric study. Cephalalgia.

[27] Fazekas F, Koch M, Schmidt R (1992). The prevalence of cerebral damage varies with migraine type: a MRI study. Headache.

[28] Cooney BS, Grossman RI, Farber RE (1996). Frequency of magnetic resonance imaging abnormalities in patients with migraine. Headache.

[29] Rocca MA, Colombo B, Pratesi A (2000). A magnetization transfer imaging study of the brain in patients with migraine. Neurology.

[30] De Benedettis G, Lorenzetti A, Sina C (1995). Magnetic resonance imaging in migraine and tension-type headache. Headache.

[31] Palm-Meinders IH, Koppen H, Terwindt GM (2012). Structural brain changes in migraine. JAMA.

[32] Swartz RH, Kern RZ (2004). Migraine is associated with magnetic resonance imaging white matter abnormalities: a meta-analysis. Arch Neurol.

